# Supplementary material for: Risk-sensitive learning is a winning strategy for leading an urban invasion
Source: eLife. 2024 Apr 2;12:RP89315. doi: 10.7554/eLife.89315 (PMC10987091; doi:10.7554/eLife.89315)
Supplement: Supplementary file 1. [file elife-89315-supp1.pdf]

This preregistration of our study, which is intended to serve as a record of our scientific method, and which necessarily evolved beyond this original outline, was uploaded on 25 January 2022 to our GitHub repository: https://github.com/alexisbreen/Sex-differences-in-grackles-learning; and to OSF on 21 February 2022: https://doi.org/10.17605/OSF.IO/V3WXB

# Investigating sex differences in learning in a range-expanding bird

Alexis J. Breen[1,*] & Dominik Deffner[1,2,3]

[1]Department of Human Behavior, Ecology and Culture, Max Planck Institute for Evolutionary Anthropology, Leipzig 04103, Germany

[2]Science of Intelligence Excellence Cluster, Technical University Berlin, Berlin 10623, Germany

[3]Center for Adaptive Rationality, Max Planck Institute for Human Development, Berlin 14195, Germany

*alexis_breen@eva.mpg.de

## Abstract

How might differences in dispersal and learning interact in range expansion dynamics? To begin to answer this question, in this preregistration we detail the background, hypothesis plus associated predictions, and methods of our proposed study, including the development and validation of a mechanistic reinforcement learning model, which we aim to use to assay colour-reward reinforcement learning (and the influence of two candidate latent parameters—speed and sampling rate—on this learning) in great-tailed grackles—a species undergoing rapid range expansion, where males disperse.

## Introduction

Dispersal and range expansion go 'hand in hand'; movement by individuals away from a population's core is a pivotal precondition of witnessed growth in species' geographic limits (Chuang & Peterson, 2016; Ronce, 2007). Because 'who' disperses—in terms of sex—varies both within and across taxa (for example, male-biased dispersal is dominant among fish and mammals, whereas female-biased dispersal is dominant among birds; see Table 1 in Trochet et al., 2016), skewed sex ratios are apt to arise at expanding range fronts, and, in turn, differentially drive invasion dynamics. Female-biased dispersal, for instance, can 'speed up' staged invertebrate invasions by increasing offspring production (Miller & Inouye, 2013). Alongside sex-biased dispersal, learning ability is also argued to contribute to species' colonisation capacity, as novel environments inevitably present novel (foraging, predation, shelter, and social) challenges that newcomers need to surmount in order to settle successfully (Sol et al., 2013; Wright et al., 2010). Indeed, a growing number of studies show support for this supposition (as recently reviewed in Lee & Thornton, 2021). Carefully controlled choice tests, for example, show that urban-dwelling individuals—that is, the 'invaders'—will both learn and unlearn novel reward-stimulus pairings more rapidly than their rural-dwelling counterparts (Batabyal & Thaker, 2019), suggesting that range expansion selects for enhanced learning ability at the dispersal and/or settlement stage(s). Given the independent influence of sex-biased dispersal and learning ability on range expansion, it is perhaps surprising, then, that their potential interactive influence on this aspect of movement ecology remains unexamined, particularly as interactive links between dispersal and other behavioural traits such as aggression are documented within the range expansion literature (Duckworth, 2006; Gutowsky & Fox, 2011).

That learning ability can covary with, for example, exploration (e.g., Auersperg et al., 2011; Guillette et

al., 2011) and neophobia (e.g., Verbeek et al., 1994), two behaviours which may likewise play a role in range expansion (Griffin et al., 2017; Lee & Thornton, 2021), is one potential reason for the knowledge gap introduced above. Such correlations stand to mask what contribution, if any, learning ability lends to range expansion—an undoubtedly daunting research prospect. A second (and not mutually exclusive) reason is that, for many species, a detailed diary of their range expansion is lacking (Blackburn et al., 2009; Udvardy & Papp, 1969). And patchy population records inevitably introduce interpretive 'noise,' imaginably impeding population comparisons of learning ability (or the like).

In range-expanding great-tailed grackles (*Quiscalus mexicanus*), however, learning ability appears to represent a unique source of individual variation; more specifically, temporarily-captive great-tailed grackles' speed to solve colour-reward reinforcement learning tests does not correlate with measures of their exploration (time spent moving within a novel environment), inhibition (time to reverse a colour-reward preference), motor diversity (number of distinct bill and/or feet movements used in behavioural tests), neophobia (latency to approach a novel object), risk aversion (time spent stationary within a 'safe spot' in a novel environment), persistence (number of attempts to engage in behavioural tests), or problem solving (number of test-relevant functional and non-functional object-choices) (Logan, 2016a, 2016b). Moreover, careful combing by researchers of public records, such as regional bird reports and museum collections, means that great-tailed grackle range-expansion data is both comprehensive and readily available (Dinsmore & Dinsmore, 1993; Pandolfino et al., 2009; Wehtje, 2003). Thus, great-tailed grackles offer behavioural ecologists a useful study system to investigate the interplay between life-history strategies, learning ability, and range expansion.

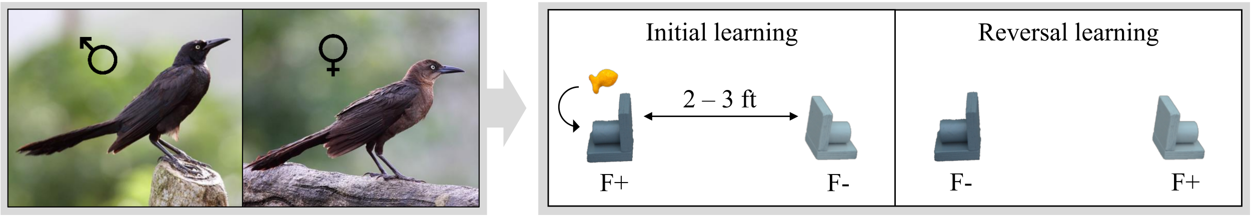


**Figure 1** Left panel: images showing a male and female great-tailed grackle (credit: Wikimedia Commons). Right panel: schematic of the colour-reward reinforcement learning experimental protocol. In the *initial learning* phase, great-tailed grackles are presented with two colour-distinct tubes; however, only one coloured tube (e.g., dark grey) contains a food reward (F+ versus F-). In the *reversal learning* phase, the colour-reward tube-pairings are swapped. The passing criterion was identical in both phases (see main text for details).

Here, for the first time (to our knowledge), we propose to investigate potential differences in colour-reward reinforcement learning performance between male and female great-tailed grackles (Figure 1), to test the hypothesis that sex differences in learning ability are related to sex differences in dispersal. Since the late nineteenth century, great-tailed grackles have been expanding their range at an unprecedented rate, moving northward from their native range in Central America into the United States (breeding in at least 20 states), with several first-sightings spanning as far north as Canada (Dinsmore & Dinsmore, 1993; Wehtje, 2003). Notably, the record of this range expansion in great-tailed grackles is heavily peppered with first-sightings involving a single or multiple male(s) (Dinsmore & Dinsmore, 1993; Kingery, 1972; Littlefield, 1983; Stepney, 1975; Wehtje, 2003). Moreover, recent genetic data show that, when comparing great-tailed grackles within a population, average relatedness: (*i*) is higher among females than among males; and (*ii*) decreases with increasing geographic distance among females; but (*iii*) is unrelated to geographic distance among males; hence, confirming a role for male-biased dispersal in great-tailed grackles (Sevchik et al., in press). Considering these natural history and genetic data, then, we expect male and female great-tailed grackles to differ across at least two colour-reward reinforcement learning parameters: speed and sampling rate (here, sampling is defined as switching between choice-options). Specifically, we expect male—versus female—great-tailed grackles: (prediction 1 & 2) to be faster to, firstly, learn a novel colour-reward pairing, and secondly, reverse their colour preference when the colour-reward pairing is swapped; and (prediction 3) to be more deterministic—that is, sample less often—in their colour-reward learning; if learning ability and dispersal relate. Indeed, since invading great-tailed grackles face agribusiness-led wildlife management

strategies, including the use of chemical crop repellents (Werner et al., 2011, 2015), range expansion should disfavour slow, error-prone learning strategies, resulting in a spatial sorting of learning ability in great-tailed grackles (Wright et al., 2010). Related to this final point, we further expect (prediction 4) such sex differences in learning ability to be more pronounced in great-tailed grackles living at the edge, rather than the intermediate and/or core, region of their range (e.g., Duckworth, 2006).

# Methods

## Data

This preregistration aims to use colour-reward reinforcement learning data collected (or being collected) in great-tailed grackles across three study sites that differ in their range-expansion demographics; that is, belonging to a core, intermediate, or edge population (based on time-since-settlement population growth dynamics, as outlined in Chuang & Peterson, 2016). Specifically, data will be utilised from: (*i*) Tempe, Arizona—hereafter, the core population (estimated—by adding the average time between first sighting and first breeding to the year first sighted—to be breeding since 1951) (Walter, 2004; Wehtje, 2003); (*ii*) Santa Barbara, California—hereafter, the intermediate population (known to be breeding since 1996) (Lehman, 2020); and (*iii*) Woodland, California—hereafter, the edge population (known to be breeding since 2004) (Hampton, 2001). Data collection at both the Tempe, Arizona and Santa Barbara, California study sites has been completed prior to the submission of this preregistration (total sample size across sites: nine females and 25 males); however, data collection at the Woodland, California study site is ongoing (current sample size: three females and nine males; anticipated minimum total sample size: five females and ten males). Thus, the final data set should contain colour-reward reinforcement learning data from at least 14 female and 35 male great-tailed grackles.

## Experimental protocol

### *General*

A step-by-step description of the experimental protocol is reported elsewhere (e.g., Blaisdell et al., 2021). As such, below we detail only the protocol for the colour-reward reinforcement learning tests that we propose to analyse herein.

### *Colour-reward reinforcement learning tests*

The reinforcement learning tests consist of two phases (Figure 1, right panel): (*i*) colour-reward learning (hereafter, initial learning) and (*ii*) colour-reward reversal learning (hereafter, reversal learning). In both phases, two different coloured tubes are used: for Santa Barbara great-tailed grackles, gold and grey (Logan, 2016b, 2016a); for all other great-tailed grackles: light and dark grey (Blaisdell et al., 2021). Each tube consists of an outer and inner diameter of 26 mm and 19 mm, respectively; and each is mounted to two pieces of plywood attached at a right angle (entire apparatus: 50 mm wide × 50 mm tall × 67 mm deep); thus resulting in only one end of each coloured tube being accessible (Figure 1, right panel).

In the *initial learning phase*, great-tailed grackles are required to learn that only one of the two coloured tubes contains a food reward (e.g., dark grey; this colour-reward pairing is counterbalanced across great-tailed grackles within each study site). Specifically, the rewarded and unrewarded coloured tubes are placed—either on a table or on the floor—in the centre of the aviary run (distance apart: table, 2 ft; floor, 3 ft), with the open tube-ends facing, and perpendicular to, their respective aviary side-wall. Which coloured tube is placed on which side of the aviary run (left or right) is pseudorandomised across trials. A trial begins at tube-placement, and ends when a great-tailed grackle has either made a tube-choice or the maximum trial time has elapsed (eight minutes). A tube-choice is defined as a great-tailed grackle bending down to examine the contents (or lack thereof) of a tube. If the chosen tube contains food, the great-tailed grackle is allowed to retrieve and eat the food, before both tubes are removed and the rewarded coloured tube is rebaited out of sight (for the great-tailed grackle). If a chosen tube does not contain food, both tubes are immediately removed. Each great-tailed grackle is given, first, up to three minutes to make a tube-choice (after which a piece of food is placed equidistant between the tubes to entice participation); and then, if no choice has been made, an additional five minutes maximum, before both tubes are removed. All trials are recorded as either correct (choosing the rewarded colour tube), incorrect (choosing the unrewarded colour tube), or

incomplete (no choice made); and are presented in 10-trial blocks. To pass initial learning, a great-tailed grackle must make a correct choice in at least 17 out of the most recent 20 trials, with a minimum of eight and nine correct choices across the last two blocks.

In the *reversal learning phase*, great-tailed grackles are required to learn that the colour-reward pairing has been switched; that is, the previously unrewarded coloured tube (e.g., light grey) now contains a food reward. The protocol for this second and final learning phase is identical to that, described above, of the initial learning phase.

## Analysis plan

### *General*

Here, we will analyse, process, and visually present our data using, respectively, the 'rstan' (Stan Development Team, 2020), 'rethinking' (McElreath, 2018), and 'tidyverse' (Wickham et al., 2019) packages in R (R Core Team, 2021). Our reproducible code is available on GitHub (https://github.com/alexisbreen/Sex-differences-in-grackles-learning).

### *Reinforcement learning model*

In this preregistration, we propose to employ an adapted (from Deffner et al., 2020) Bayesian reinforcement learning model, to examine the influence of sex on great-tailed grackles' initial and reversal learning performance. The reinforcement learning model, defined below, allows us to link observed coloured tube-choices to latent individual-level knowledge-updating (of attractions towards, learning about, and sampling of, either coloured tube) based on recent tube-choice reward-payoffs, and to translate such latent knowledge-updating into individual tube-choice probabilities; in other words, we can reverse engineer the probability that our parameters of interest (speed and sampling rate) produce great-tailed grackles' observed tube-choice behaviour by formulating our scientific model as a statistical model (McElreath, 2018, p. 537). This method can therefore capture whether, and, if so, how multiple latent learning strategies simultaneously guide great-tailed grackles' decision making—an analytical advantage over more traditional methods (e.g., comparing trials to passing criterion) that ignore the potential for equifinality (Barrett, 2019; Kandler & Powell, 2018).

Our reinforcement learning model consists of two equations:

$$A_{i,j,t+1} = (1 - \phi_{k,l})A_{i,j,t} + \phi_{k,l}\pi_{i,j,t}, \tag{1}$$

$$P(i)_{t+1} = \frac{\exp(\lambda_{k,l}A_{i,j,t})}{\sum\limits_{m=1}^{2}\exp(\lambda_{k,l}A_{m,j,t})}. \tag{2}$$

Equation 1 expresses how attraction ($A$) to a choice-option ($i$) changes for an individual ($j$) across time ($t+1$) based on their prior attraction to that choice-option ($A_{i,j,t}$) plus their recently experienced choice-payoff ($\pi_{i,j,t}$), whilst accounting for the weight given to recent payoffs ($\phi_{k,l}$). As $\phi_{k,l}$ increases in value, so, too, does the rate of individual attraction-updating; thus, $\phi_{k,l}$ represents the individual learning rate. We highlight that the $k, l$ indexing denotes that we estimate separate $\phi$ parameters for each phase of the experiment ($k = 1$ for initial, $k = 2$ for reversal) and each sex ($l = 1$ for females, $l = 2$ for males).

Equation 2 is a softmax function that expresses the probability ($P$) that option ($i$) is selected in the next choice-round ($t+1$) as a function of the attractions and a parameter ($\lambda_{k,l}$) that governs how much relative differences in attraction scores guide individual choice-behaviour. The higher the value of $\lambda_{k,l}$, the more deterministic (less option-switching) the choice-behaviour of an individual becomes (note $\lambda_{k,l} = 0$ generates random choice); thus, $\lambda_{k,l}$ represents the individual sampling rate for phase $k$ and sex $l$.

From the above reinforcement learning model, then, we will generate inferences about the effect of sex on $\phi_{k,l}$ and $\lambda_{k,l}$ from at least 1000 effective samples of the posterior distribution (see our model validation below). We note that our reinforcement learning model also includes both individual bird and study site as random effects (to account for repeated measures within both individuals and populations); however, for clarity,

these parameters are omitted from our equations (but not our code: https://github.com/alexisbreen/Sex-differences-in-grackles-learning). Regarding our study site random effect, we further note that, as introduced above, we will also explore population-mediated sex-effects on $\phi$ and $\lambda$, by comparing these learning parameters both within and between sexes at each study site. Finally, our reinforcement learning model excludes trials where a great-tailed grackle did not make a tube-choice, as this measure cannot clearly speak to individual learning ability—for example, satiation rather than any learning of 'appropriate' colour tube-choice could be invoked as an explanation in such cases. Indeed, there are, admittedly, a number of intrinsic and extrinsic factors (e.g., temperament and temperature, respectively) that might bias great-tailed grackles' tube-choice behaviour, and, in turn, the output from our reinforcement learning model (Webster & Rutz, 2020). Nonetheless, our reinforcement learning model serves as a useful first step towards addressing if learning ability and dispersal relate in great-tailed grackles (for a similiar rationale, see McElreath & Smaldino, 2015).

*Model validation*

We validated our reinforcement learning model in three steps. First, we performed agent-based simulations. Specifically, we followed the tube-choice behaviour of simulated great-tailed grackles—that is, 14 females and 35 males from one of three populations (where population membership matched known study site sex distributions)—across the described initial learning and reversal learning phases. The tube-choice behaviour of the simulated great-tailed grackles was governed by a set of rules identical to those defined by our mathematical equations—for example, coloured tube attractions were independently updated based on the reward outcome of tube choices. Because we assigned higher average $\phi$ and $\lambda$ values to simulated male (versus female) great-tailed grackles, the resulting data set should show males outperform females on initial and reversal learning, at both the group and individual-level; it did (Figure 2 & S1, respectively).

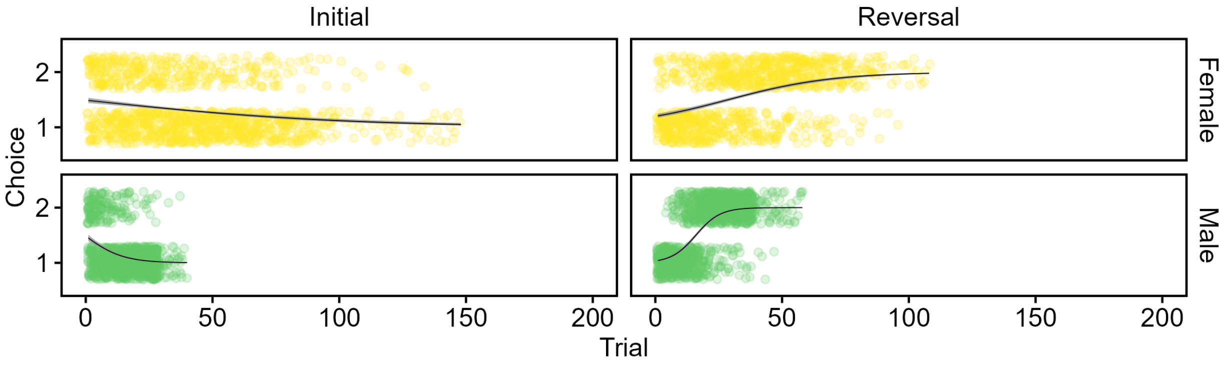


**Figure 2** Group-level tube-choice behaviour of simulated great-tailed grackles across colour-reward reinforcement learning trials (females: yellow, $n = 14$; males: green, $n = 35$), following model validation step one. Tube option 1 (e.g., dark grey) was the rewarded option in the initial learning phase; conversely, tube option 2 (e.g., light grey) contained the food reward in the reversal learning phase. Each open circle represents an individual tube-choice; black lines indicate binomial smoothed conditional means fitted with grey 89% compatability intervals.

Next, we ran our simulated data set on our reinforcement learning model. Here, we endeavored to determine whether our reinforcement learning model: (*i*) recovered our assigned $\phi_{k,l}$ and $\lambda_{k,l}$ values (it did; Table 1); and (*ii*) produced 'correct' qualitative inferences—that is, detected the simulated sex differences in great-tailed grackles' initial and reversal learning (it did; Figure 3).

Table 1: Comparison of assigned and recovered $\phi$ and $\lambda$ values, following model validation step two. Eighty-nine percent highest posterior density intervals (HPDI) are shown for recovered values.

| | $\phi$ | | | | $\lambda$ | | | |
|---|---|---|---|---|---|---|---|---|
| | Female | | Male | | Female | | Male | |
| | Initial | Reversal | Initial | Reversal | Initial | Reversal | Initial | Reversal |
| Assigned | 0.03 | 0.05 | 0.09 | 0.11 | 2.00 | 3.00 | 4.00 | 5.00 |
| Recovered | 0.03 | 0.05 | 0.07 | 0.10 | 2.16 | 2.82 | 4.31 | 5.68 |
| 89% HPDI | 0.01 - 0.04 | 0.04 - 0.06 | 0.03 - 0.11 | 0.08 - 0.12 | 1.29 - 2.99 | 2.05 - 3.58 | 2.65 - 6.00 | 4.41 - 6.97 |

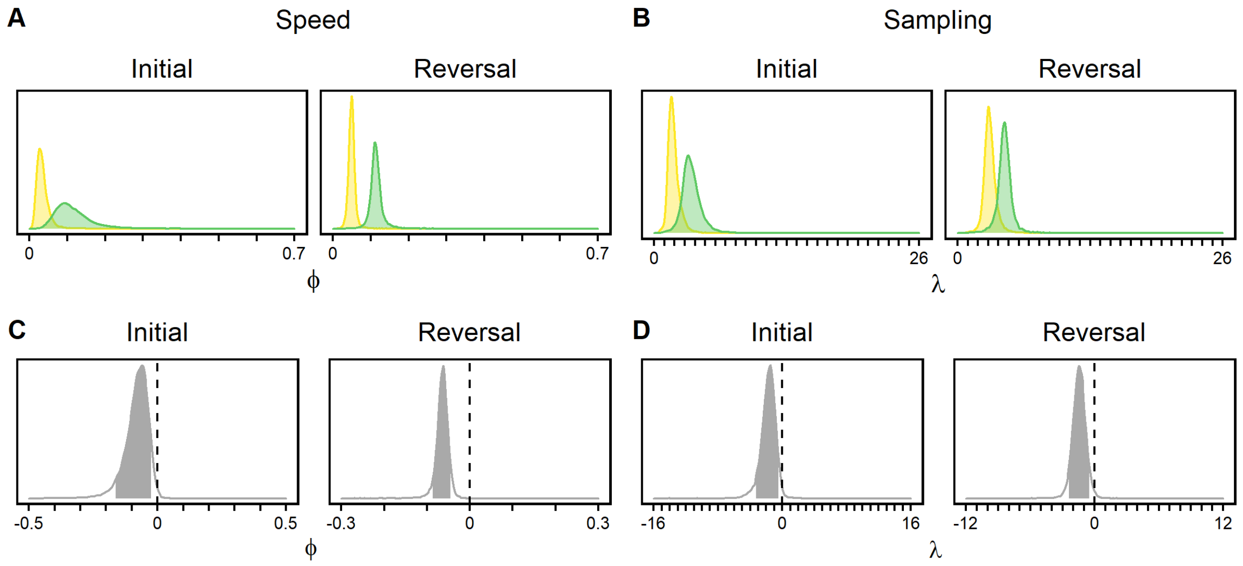


**Figure 3** Comparison of learning ability in simulated female (yellow; $n = 14$) and male (green; $n = 35$) great-tailed grackles across initial and reversal colour-reward reinforcement learning, following model validation step two. (**A**) $\phi$, the rate of learning i.e., speed. (**B**) $\lambda$, the rate of sampling i.e., switching between choice-options. (**C**) and (**D**) show posterior distributions for respective contrasts between female and male learning. Eighty-nine percent highest posterior density intervals are shaded in grey; that this interval does not cross zero evidences a simulated effect of sex on learning ability.

Finally, we repeated step one and step two, using a range of realistically plausible $\phi$ and $\lambda$ sex differences (note that values for female great-tailed grackles were left unchanged from Table 1), to determine whether our reinforcement learning model could detect different effect sizes of sex on our target learning parameters. This final step confirmed that, for our anticipated minimum sample size, our reinforcement learning model: (*i*) detects sex differences in $\phi$ values $>= 0.03$ and $\lambda$ values $>= 1$; and (*ii*) infers a null effect for $\phi$ values $< 0.03$ and $\lambda$ values $< 1$ i.e., very weak simulated sex differences (Figure 4). Both of these points together highlight how our reinforcement learning model allows us to say that null results are not just due to small sample size. Additionally, estimates obtained from step three were more precise in the reversal learning phase compared to the initial learning phase (Figure 4), and we can expect to detect even smaller sex differences if we analyse learning across both phases—an approach we will apply if we detect no effect of phase. In sum, model validation steps one through three confirm that our reinforcement learning model is reasonably fit.

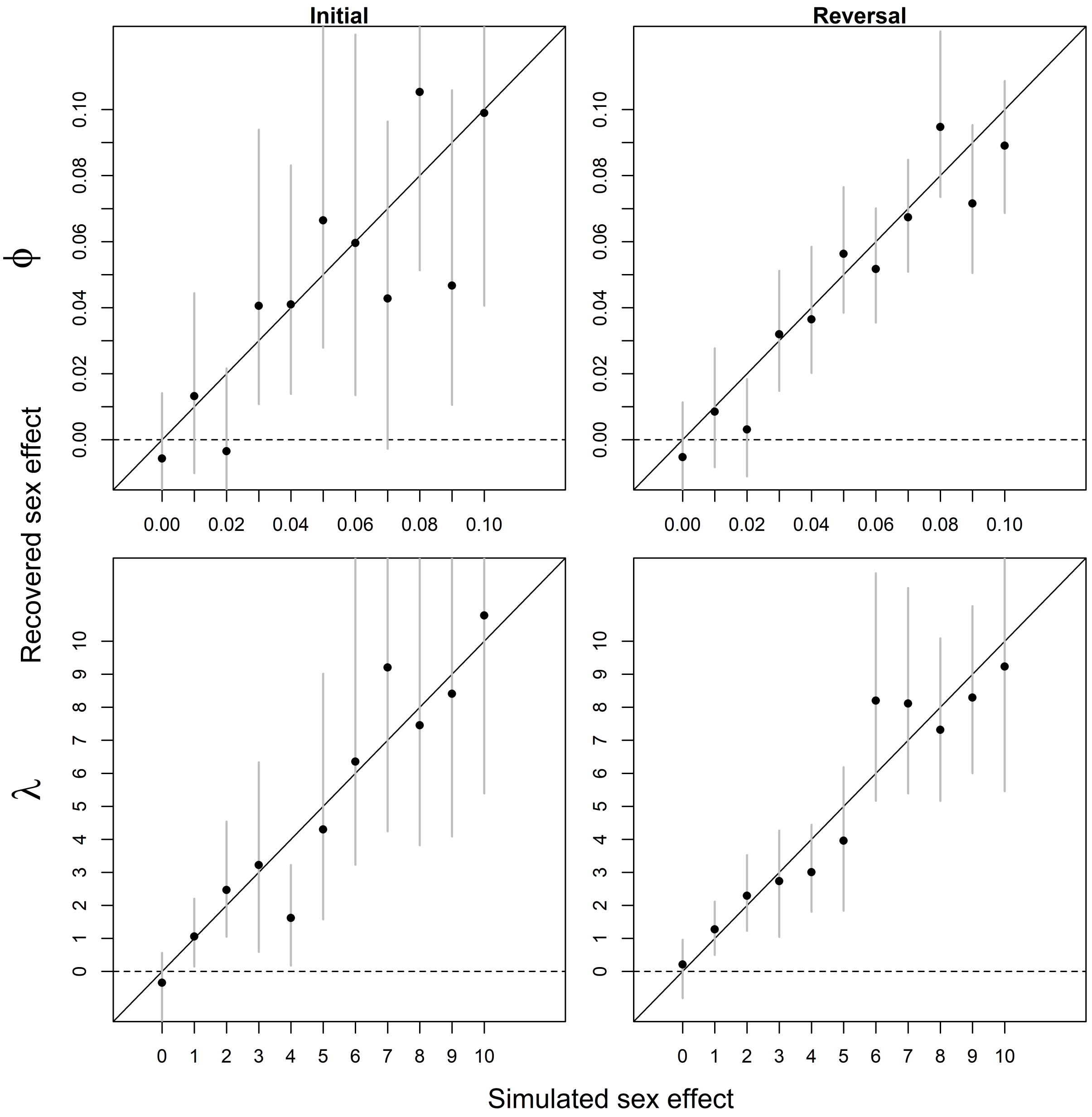


**Figure 4** Parameter recovery test for different sizes of simulated sex differences. Plots show posterior estimates of the effect of sex (contrasts between simulated male and female great-tailed grackles; $n$ = 14 and 35, respectively) on speed ($\phi$) and sampling ($\lambda$) learning parameters, following model validation step three. Black circles represent the mean recovered sex effect estimates with grey eighty-nine percent highest posterior density intervals (HPDIs); black solid diagonal lines represent a 'perfect' match between assigned and recovered parameter estimates (note that we would not expect a perfect correspondence due to stochasticity of agent-based simulations); and black dashed horizontal lines represent a recovered null sex effect.

## Bias

AJB and DD are (at the time of submitting this preregistration) blind with respect to all but two aspects of the target data: the sex and population membership of each grackle that has, thus far, completed, or is expected to complete, the colour-reward reinforcement learning tests (because these parameters were used in model validation simulations—see above).

## Open materials

https://github.com/alexisbreen/Sex-differences-in-grackles-learning


## Acknowledgements

We thank all members, past and present, of the Grackle Project for collecting and sharing the data that we propose to analyse herein. We further thank Richard McElreath for study support.


## Ethics

All data utilised herein were collected with ethical approval.

## References


Auersperg, A. M. I., Von Bayern, A. M. P., Gajdon, G. K., Huber, L., & Kacelnik, A. (2011). Flexibility in problem solving and tool use of kea and New Caledonian crows in a multi access box paradigm. *PloS ONE*, *6*(6), e20231. https://doi.org/10.1371/journal.pone.0020231

Barrett, B. J. (2019). Equifinality in empirical studies of cultural transmission. *Behavioural Processes*, *161*, 129–138. https://doi.org/10.1016/j.beproc.2018.01.011

Batabyal, A., & Thaker, M. (2019). Lizards from suburban areas learn faster to stay safe. *Biology Letters*, *15*(2), 20190009. https://doi.org/10.1098/rsbl.2019.0009

Blackburn, T. M., Lockwood, J. L., & Cassey, P. (2009). *Avian invasions: The ecology and evolution of exotic birds* (Vol. 1). Oxford University Press.

Blaisdell, A., Seitz, B., Rowney, C., Folsom, M., MacPherson, M., Deffner, D., & Logan, C. J. (2021). Do the more flexible individuals rely more on causal cognition? Observation versus intervention in causal inference in great-tailed grackles. *Peer Community Journal*, *1*(e50). https://doi.org/10.24072/pcjournal.44

Chuang, A., & Peterson, C. R. (2016). Expanding population edges: Theories, traits, and trade-offs. *Global Change Biology*, *22*(2), 494–512. https://doi.org/10.1111/gcb.13107

Deffner, D., Kleinow, V., & McElreath, R. (2020). Dynamic social learning in temporally and spatially variable environments. *Royal Society Open Science*, *7*(12), 200734. https://doi.org/10.1098/rsos.200734

Dinsmore, J. J., & Dinsmore, S. J. (1993). Range expansion of the great-tailed grackle in the 1900s. *Journal of the Iowa Academy of Science*, *100*(2), 54–59.

Duckworth, R. A. (2006). Behavioral correlations across breeding contexts provide a mechanism for a cost of aggression. *Behavioral Ecology*, *17*(6), 1011–1019. https://doi.org/10.1093/beheco/arl035

Griffin, A. S., Netto, K., & Peneaux, C. (2017). Neophilia, innovation and learning in an urbanized world: A critical evaluation of mixed findings. *Current Opinion in Behavioral Sciences*, *16*, 15–22. https://doi.org/10.1016/j.cobeha.2017.01.004

Guillette, L. M., Reddon, A. R., Hoeschele, M., & Sturdy, C. B. (2011). Sometimes slower is better: Slow-exploring birds are more sensitive to changes in a vocal discrimination task. *Proceedings of the Royal Society B: Biological Sciences*, *278*(1706), 767–773. https://doi.org/10.1098/rspb.2010.1669

Gutowsky, L. F. G., & Fox, M. G. (2011). Occupation, body size and sex ratio of round goby *Neogobius melanostomus* in established and newly invaded areas of an ontario river. *Hydrobiologia*, *671*(1), 27–37. https://doi.org/10.1007/s10750-011-0701-9

Hampton, S. (2001). *Yolo County birding news.*

Kandler, A., & Powell, A. (2018). Generative inference for cultural evolution. *Philosophical Transactions of the Royal Society B: Biological Sciences*, *373*(1743), 20170056. https://doi.org/10.1098/rstb.2017.0056

Kingery, H. E. (1972). The nesting season: June 1-August 15, 1972. *American Birds*, *26*, 882–887.

Lee, V. E., & Thornton, A. (2021). Animal cognition in an urbanised world. *Frontiers in Ecology and Evolution*, *9*, 120. https://doi.org/10.3389/fevo.2021.633947

Lehman, P. E. (2020). *The birds of Santa Barbara County, California* (2nd ed.). https://doi.org/http://www.sbcobirding.com/lehmanbosbc.html

Littlefield, C. D. (1983). Oregon's first records of the great-tailed grackle. *Western Birds*, *14*, 201–202.

Logan, C. J. (2016a). Behavioral flexibility and problem solving in an invasive bird. *PeerJ*, *4*, e1975. https://doi.org/10.7717/peerj.1975

Logan, C. J. (2016b). Behavioral flexibility in an invasive bird is independent of other behaviors. *PeerJ*, *4*, e2215. https://doi.org/10.7717/peerj.2215

McElreath, R. (2018). *Statistical rethinking: A Bayesian course with examples in R and Stan*. Chapman; Hall/CRC.

McElreath, R., & Smaldino, P. E. (2015). Replication, communication, and the population dynamics of scientific discovery. *PloS One*, *10*(8), e0136088. https://doi.org/10.1371/journal.pone.0136088

Miller, T. E. X., & Inouye, B. D. (2013). Sex and stochasticity affect range expansion of experimental invasions. *Ecology Letters*, *16*(3), 354–361. https://doi.org/10.1111/ele.12049

Pandolfino, E. R., Deuel, B. E., & Young, L. (2009). Colonization of the california's central valley by the great-tailed grackle. *Central Valley Bird Club Bull*, *12*, 77–95.

R Core Team. (2021). *R: A language and environment for statistical computing* (Version 1.4.1106) [Computer software]. https://www.R-project.org/

Ronce, O. (2007). How does it feel to be like a rolling stone? Ten questions about dispersal evolution. *Annual Review of Ecology, Evolution, and Systematics*, *38*, 231–253. https://doi.org/10.1146/annurev.ecolsys.38.091206.095611

Sevchik, A., Logan, C. J., McCune, K. B., Blackwell, A., Rowney, C., & Lukas, D. (in press). Investigating sex differences in genetic relatedness in great-tailed grackles in Tempe, Arizona to infer potential sex biases in dispersal. In *Peer community in ecology*. https://doi.org/10.32942/osf.io/t6beh

Sol, D., Lapiedra, O., & González-Lagos, C. (2013). Behavioural adjustments for a life in the city. *Animal Behaviour*, *85*(5), 1101–1112. https://doi.org/10.1016/j.anbehav.2013.01.023

Stan Development Team. (2020). *RStan: The R interface to Stan* (Version 2.21.2) [Computer software]. http://mc-stan.%20org

Stepney, P. H. R. (1975). First recorded breeding of the great-tailed grackle in Colorado. *The Condor*, *77*(2), 208–210. https://doi.org/10.2307/1365794

Trochet, A., Courtois, E. A., Stevens, V. M., Baguette, M., Chaine, A., Schmeller, D. S., Clobert, J., & Wiens, J. J. (2016). Evolution of sex-biased dispersal. *The Quarterly Review of Biology*, *91*(3), 297–320. https://doi.org/10.1086/688097

Udvardy, M. D. F., & Papp, C. S. (1969). *Dynamic zoogeography*. Van Nostrand Reinhold.

Verbeek, M. E. M., Drent, P. J., & Wiepkema, P. R. (1994). Consistent individual differences in early exploratory behaviour of male great tits. *Animal Behaviour*, *48*(5), 1113–1121. https://doi.org/10.1006/anbe.1994.1344

Walter, W. (2004). *The great-tailed grackle* (Quiscalus mexicanus *Gmelin) in the Western USA: Range expansion and secondary contact between subspecies* [PhD thesis]. University of California Riverside.

Webster, M. M., & Rutz, C. (2020). How STRANGE are your study animals? In *Nature* (No. 7812; Vol. 582, pp. 337–340). Nature Publishing Group. https://doi.org/10.1038/d41586-020-01751-5

Wehtje, W. (2003). The range expansion of the great-tailed grackle *(Quiscalus mexicanus* Gmelin) in North America since 1880. *Journal of Biogeography*, *30*(10), 1593–1607. https://doi.org/10.1046/j.1365-2699.2003.00970.x

Werner, S. J., DeLiberto, S. T., Mangan, A. M., Pettit, S. E., Ellis, J. W., & Carlson, J. C. (2015). Anthraquinone-based repellent for horned larks, great-tailed grackles, American crows and the protection of California's specialty crops. *Crop Protection*, *72*, 158–162. https://doi.org/10.1016/j.cropro.2015.03.020

Werner, S. J., Linz, G. M., Carlson, J. C., Pettit, S. E., Tupper, S. K., & Santer, M. M. (2011). Anthraquinone-based bird repellent for sunflower crops. *Applied Animal Behaviour Science*, *129*(2-4), 162–169. https://doi.org/10.1016/j.applanim.2010.11.010

Wickham, H., Averick, M., Bryan, J., Chang, W., McGowan, L. D., François, R., Grolemund, G., Hayes, A., Henry, L., Hester, J., Kuhn, M., Pedersen, T. L., Miller, E., Bache, S. M., Müller, l, Ooms, J., Robinson, D., Seidel, D. P., Spinu, V., … Yutani, H. (2019). Welcome to the Tidyverse. *Journal of Open Source Software*, *4*(43), 1686. https://doi.org/10.21105/joss.01686

Wright, T. F., Eberhard, J. R., Hobson, E. A., Avery, M. L., & Russello, M. A. (2010). Behavioral flexibility and species invasions: The adaptive flexibility hypothesis. *Ethology Ecology & Evolution*, *22*(4), 393–404. https://doi.org/10.1080/03949370.2010.505580

**Supplementary material**

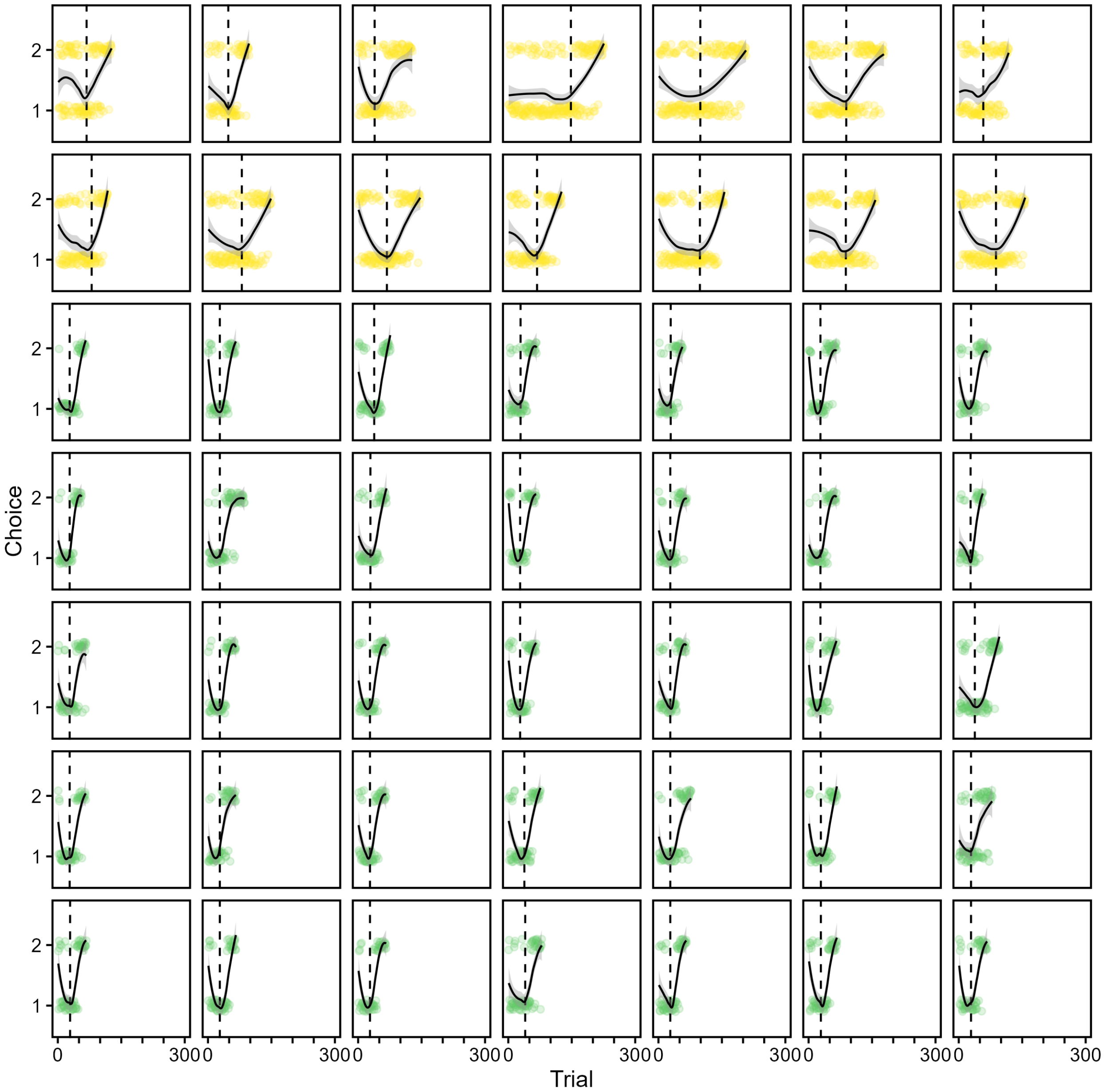


**Figure S1** Individual-level tube-choice behaviour of simulated great-tailed grackles across colour-reward reinforcement learning trials (females: yellow, $n = 14$; males: green, $n = 35$). Tube option 1 (e.g., dark grey) was the rewarded option in the initial learning phase; conversely, tube option 2 (e.g., light grey) contained the food reward in the reversal learning phase. Each open circle shows an individual tube-choice; black solid lines show loess smoothed conditional means fitted with grey 89% compatibility intervals; and dashed black lines show individual-unique transitions between learning phases.
