# Supplementary material for: Risk-sensitive learning is a winning strategy for leading an urban invasion
Source: eLife. 2024 Apr 2;12:RP89315. doi: 10.7554/eLife.89315 (PMC10987091; doi:10.7554/eLife.89315)
Supplement: Supplementary file 2. — (a) Total-trials-in-test Poisson regression model output. (b) Total-choice-option-switches-in-test Poisson regression model output. (c) Bayesian reinforcement learning model information-updating rate φ output. (d) Bayesian reinforcement learning model risk-sensitivity rate λ output. For (a–d), both between- and across-population posterior means and corresponding 89% highest-posterior density intervals are reported for males, females, and male-female contrasts. [file elife-89315-supp2.docx]

**Table a. Reinforcement learning speed.** Between- and across-population total-trials-in-test Poisson regression model estimates and male-female contrasts, with corresponding lower (L) and upper (U) 89% highest-posterior density intervals in parentheses.

# Initial learning

|  | Population | Male | Female | Contrast |  |
| --- | --- | --- | --- | --- | --- |
|  | Core Middle Edge Across | 36.43 (L: 31.60; U: 41.31)  27.00 (L: 19.62; U: 33.61)  33.85 (L: 28.21; U: 39.15)  33.77 (L: 30.41; U: 37.07) | 37.38 (L: 28.74; U: 45.92)  27.37 (L: 19.83; U: 34.22)  38.65 (L: 31.21; U: 45.59)  35.08 (L: 30.14; U: 39.69) | -0.95 (L: -11.09; U: 8.99)  -0.37 (L: -10.78; U: 9.79)  -4.80 (L: -13.84; U: 4.28)  -1.31 (L: -6.78; U: 4.79) |  |
|  |  |  | **Reversal learning** |  |  |
|  | Population | Male | Female | Contrast |  |
|  | Core Middle Edge Across | 61.60 (L: 53.60; U: 69.57)  78.78 (L: 58.56; U: 98.84)  66.30 (L: 56.63; U: 76.64)  60.00 (L: 54.25; U: 65.53) | 80.83 (L: 62.32; U: 97.47)  91.35 (L: 69.04; U: 111.88)  71.80 (L: 58.87; U: 84.46)  78.18 (L: 68.26; U: 88.16) | -19.22 (L: -38.46; U: 0.65)  -12.57 (L: -41.64; U: 17.50)  -5.51 (L: -21.95; U: 10.95)  -18.21 (L: -29.51; U: -6.99) |  |

**Table b. Reinforcement learning switches.** Between- and across-population total-choice-option-switches-in-test Poisson regression model estimates and male-female contrasts, with corresponding lower (L) and upper (U) 89% highest-posterior density intervals in parentheses.

# Initial learning

|  | Population | Male | Female | Contrast |  |
| --- | --- | --- | --- | --- | --- |
|  | Core Middle Edge Across | 13.64 (L: 10.69; U: 16.34)  6.52 (L: 3.35; U: 9.15)  12.81 (L: 9.54; U: 16.08)  13.23 (L: 11.07; U: 15.18) | 16.04 (L: 10.17; U: 21.59)  6.25 (L: 3.39; U: 9.15)  14.70 (L: 10.26; U: 18.72)  14.40 (L: 11.48; U: 17.31) | -2.40 (L: -8.54; U: 4.38)  0.27 (L: -4.18; U: 4.33)  -1.89 (L: -7.20; U: 3.74)  -1.17 (L: -4.72; U: 2.46) |  |
|  |  |  | **Reversal learning** |  |  |
|  | Population | Male | Female | Contrast |  |
|  | Core Middle Edge Across | 22.38 (L: 17.88; U: 26.86)  30.32 (L: 17.84; U: 42.10)  21.71 (L: 16.58; U: 27.22)  22.18 (L: 18.72; U: 25.27) | 37.02 (L: 23.96; U: 48.74)  43.96 (L: 27.82; U: 59.76)  27.75 (L: 20.10; U: 35.35)  36.74 (L: 29.78; U: 43.60) | -14.64 (L: -27.59; U: -1.17)  -13.65 (L: -34.30; U: 7.69)  -6.04 (L: -15.54; U: 3.39)  -14.56 (L: -22.48; U: -7.15) |  |

**Table c. Reinforcement learning information-updating rate ϕ.** Between- and across-population computational model ϕ estimates and male-female contrasts, with posterior means and corresponding lower (L) and upper (U) 89% highest-posterior density intervals in parentheses.

# Initial learning

|  | Population | Male | Female | Contrast |  |
| --- | --- | --- | --- | --- | --- |
|  | Core Middle Edge Across | 0.03 (L: 0.01; U: 0.05)  0.12 (L: 0.03; U: 0.20)  0.07 (L: 0.02; U: 0.11)  0.03 (L: 0.01; U: 0.04) | 0.07 (L: 0.02; U: 0.12)  0.10 (L: 0.03; U: 0.17)  0.09 (L: 0.03; U: 0.14)  0.05 (L: 0.02; U: 0.08) | -0.04 (L: -0.10; U: 0.02)  0.02 (L: -0.13; U: 0.15)  -0.02 (L: -0.10; U: 0.07)  -0.02 (L: -0.06; U: 0.01) |  |
|  |  |  | **Reversal learning** |  |  |
|  | Population | Male | Female | Contrast |  |
|  | Core Middle Edge Across | 0.03 (L: 0.02; U: 0.05)  0.04 (L: 0.02; U: 0.06)  0.04 (L: 0.03; U: 0.05)  0.03 (L: 0.01; U: 0.04) | 0.04 (L: 0.02; U: 0.05)  0.04 (L: 0.02; U: 0.06)  0.04 (L: 0.03; U: 0.06)  0.03 (L: 0.02; U: 0.07) | 0.00 (L: -0.02; U: 0.02)  0.00 (L: -0.03; U: 0.03)  0.00 (L: -0.03; U: 0.02)  0.00 (L: -0.01; U: -0.01) |  |

**Table d. Reinforcement learning risk-sensitivity rate λ.** Between- and across-population computational model λ estimates and male-female contrasts, with posterior means and corresponding lower (L) and upper (U) 89% highest posterior density intervals in parentheses.

# Initial learning

|  | Population | Male | Female | Contrast |  |
| --- | --- | --- | --- | --- | --- |
|  | Core Middle Edge Across | 4.61 (L: 2.24; U: 6.84)  3.58 (L: 1.33; U: 5.86)  2.81 (L: 1.44; U: 4.14)  5.65 (L: 3.15; U: 8.22) | 2.65 (L: 0.93; U: 4.20)  3.53 (L: 1.51; U: 5.66)  2.03 (L: 1.06; U: 2.89)  3.30 (L: 1.68; U: 4.73) | 1.96 (L: -1.06; U: 5.12)  0.00 (L: -3.43; U: 3.52)  0.79 (L: -1.19; U: 2.34)  2.35 (L: -0.77; U: 5.61) |  |
|  |  |  | **Reversal learning** |  |  |
|  | Population | Male | Female | Contrast |  |
|  | Core Middle Edge Across | 4.76 (L: 2.86; U: 6.33)  2.99 (L: 1.35; U: 4.40)  4.13 (L: 2.62; U: 5.75)  5.86 (L: 3.30; U: 8.37) | 2.20 (L: 1.10; U: 3.28)  1.74 (L: 0.80; U: 2.57)  3.62 (L: 2.12; U: 4.94)  3.50 (L: 1.98; U: 4.91) | 2.56 (L: 0.59; U: 4.82)  1.25 (L: -0.64; U: 3.12)  0.51 (L: -1.68; U: 2.67)  2.36 (L: 0.14; U: 4.26) |  |
